# Consumer Food Purchases After Glucagon-Like Peptide-1 Receptor Agonist Initiation

**DOI:** 10.1001/jamanetworkopen.2025.55449

**Published:** 2026-01-26

**Authors:** Kathrine Kold Sørensen, Frederik Trier Møller, Puriya Daniel Würtz Yazdanfard, Rasmus Hasselbalch, Kristian Hay Kragholm, Mikkel Porsborg Andersen, Christian Torp-Pedersen

**Affiliations:** 1 Department for Clinical and Translational Research, Copenhagen University Hospital–Steno Diabetes Center Copenhagen, Herlev, Denmark; 2 Department of Infectious Disease Epidemiology and Prevention, Statens Serum Institut, Copenhagen, Denmark; 3 Department of Cardiology, Copenhagen University Hospital–Herlev and Gentofte Hospital, Copenhagen, Denmark; 4 Department of Cardiology, Copenhagen University Hospital–Rigshospitalet, Copenhagen, Denmark; 5 Prehospital Center, Region Zealand, Næstved, Denmark; 6 Department of Health Science and Technology, Aalborg University, Aalborg, Denmark

## Abstract

This cross-sectional study examines whether initiating glucagon-like peptide-1 receptor agonists (GLP-1RAs) for treatment is associated with changes in the nutritional quality and processing level of supermarket purchases.

## Introduction

Glucagon-like peptide-1 receptor agonists (GLP-1RAs) are increasingly used for type 2 diabetes and obesity treatment.^1^ Their effects on appetite and satiety are well established,^2^ but less is known about their associations with food purchases. Case reports and small observational studies suggest that GLP-1RA initiation is associated with altered preferences from highly processed, energy-dense products to minimally processed foods.^3^ We examined whether initiation of GLP-1RAs for treatment was associated with changes in nutritional quality and processing level of supermarket purchases.

## Methods

This cross-sectional study used the SMIL (Health, Food, Purchases and Lifestyle) cohort,^4^ which, as of January 1, 2023, included 13 565 individuals donating supermarket receipts covering approximately 70% of Danish markets. Data were collected via a smartphone application, with some individuals contributing receipts since 2018. New GLP-1RA users (Anatomical Therapeutic Chemical code A10BJ) between 2019 and 2022 were identified through the Danish Prescription Registry and matched 1:3 with noninitiators on sex, age, and 2017 equivalized income by quartiles. To ensure no difference in sample composition, the population was restricted to individuals spending at least kr100 (to convert to US dollars, multiply by 0.1575) on edible items in both the 365 days before and after their first prescription or matched date. Individual items were categorized into nutrition-related groups using a published algorithm.^5^ We compared mean energy density (in kilocalories per 100 grams) and nutrient composition (sugar, carbohydrates, saturated fat, and protein in grams per 100 grams) across the 2 periods and classified processing level according to the NOVA system (unprocessed, minimally processed, processed, and ultraprocessed foods).^6^ Mean values were compared using *t* tests, and proportions were compared using χ^2^ tests. Analyses used R, version 4.4.1. *P* values were from 2-sided tests and deemed statistically significant at *P* < .05. Under the Danish Data Protection Act, registry-based research does not require patient informed consent or ethics approval. Data access was authorized by the Capital Region of Denmark according to the General Data Protection Regulation. Analyses were conducted from August 2018 to December 2022 on secure servers at Statistics Denmark.

## Results

**Figure 1.**
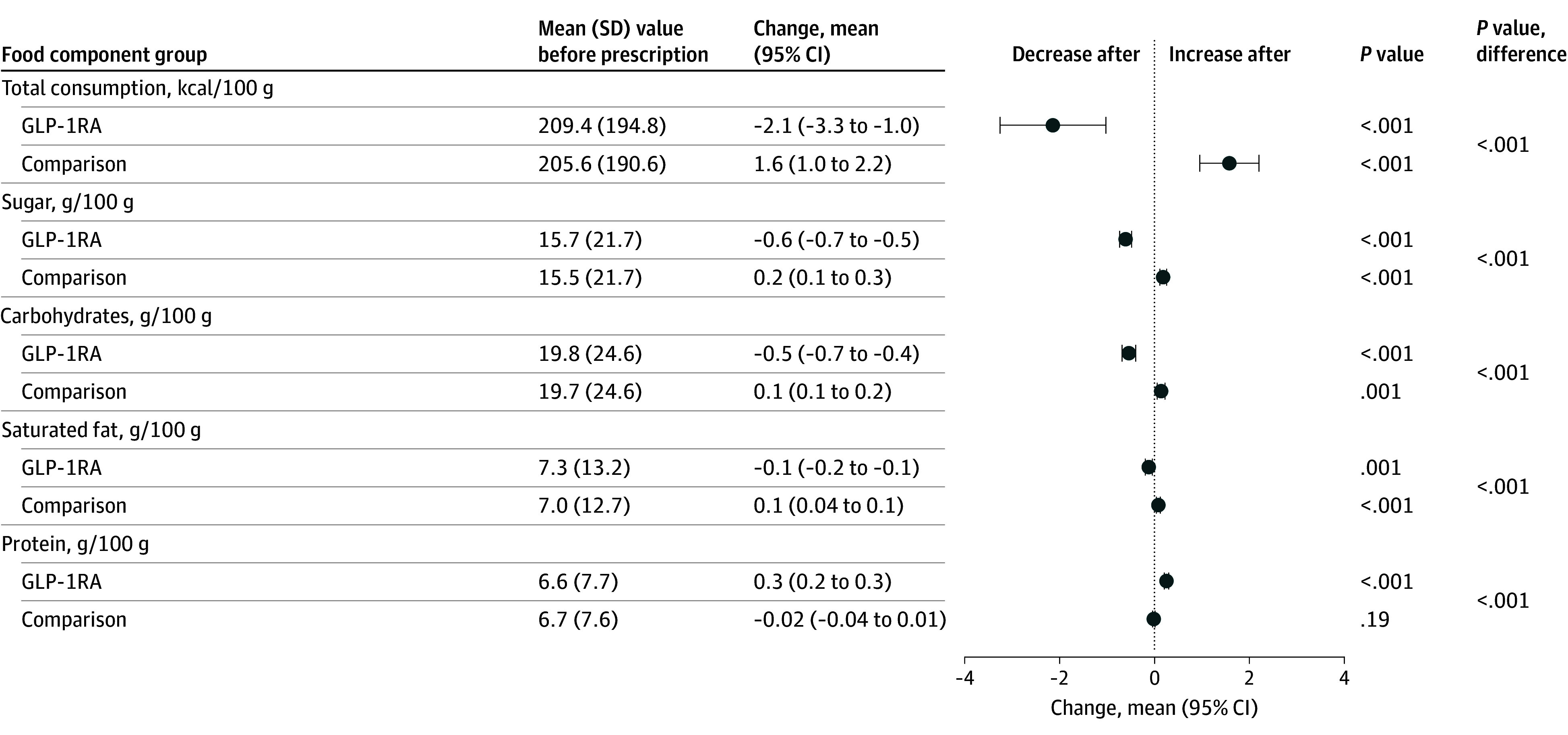
Differences in Nutrient-Related Groups Mean (SD) food components per 100 g before first glucagon-like peptide-1 receptor agonist (GLP-1RA) prescription or matched date redemption and mean change after prescription.

**Figure 2.**
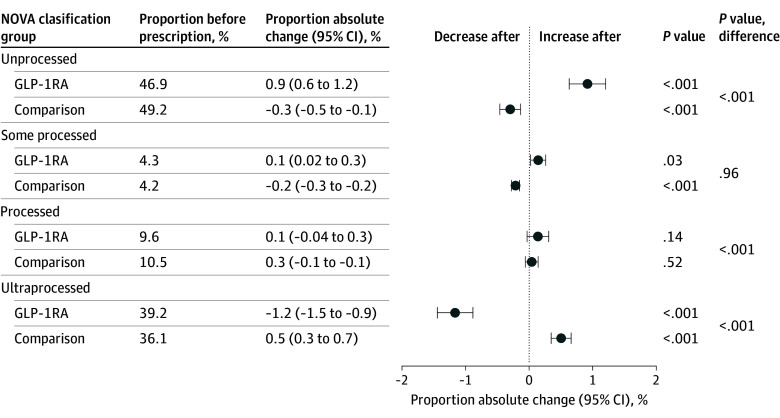
Differences in NOVA Classification Proportion of edible items classified according to the NOVA classification before first glucagon-like peptide-1 receptor agonist (GLP-1RA) prescription or matched date and difference in percentage point after prescription.

The 1177 participants (median age, 53 years [IQR, 45-61 years]; 618 women [52.5%] and 559 men [47.5%]) comprised 293 GLP-1RA initiators and 884 matched comparisons. These participants were identified from 13 565 receipt donors, of whom 453 filled at least 1 GLP-1RA prescription from 2019 to 2022. Participants spent a mean (SD) of kr52 523 (kr61 562) on edible items in the year before GLP-1 RA initiation and a mean (SD) of kr35 051 (kr47 980) the following year. In total, 807 275 purchases were analyzed before initiation and 1 171 670 after. Among GLP-1RA initiators, mean energy density decreased from 209.4 to 207.3 kcal/100 g (mean change, −2.1 [95% CI, −3.3 to −1.0] kcal/100 g), sugar from 15.7 to 15.1 g/100 g (mean change, −0.6 [95% CI, −0.7 to −0.5] g/100 g), carbohydrates from 19.8 to 19.3 g/100 g (mean change, −0.5 [95% CI, −0.7 to −0.4] g/100 g), and saturated fat from 7.3 to 7.2 g/100 g (mean change, −0.1 [95% CI, −0.2 to −0.1] g/100 g), while protein increased from 6.6 to 6.9 g/100 g (mean change, 0.3 [95% CI, 0.2-0.3] g/100 g) (Figure 1). We also observed a shift from ultraprocessed to unprocessed goods (Figure 2). In most cases, comparisons showed opposite patterns.

## Discussion

Changes in purchasing patterns after GLP-1RA initiation were seen across most nutrient categories. Opposed to comparisons, after the first prescription, participants purchased fewer calories, sugars, saturated fats, and carbohydrates, alongside modestly more protein. The share of ultraprocessed foods also decreased. Although modest at the individual level, these changes may accumulate at the population level, particularly given increasing GLP-1RA use.

Limitations include that participants likely reflect a self-selected group willing to share receipts. Lack of body mass index information is another limitation, and observed changes may partly reflect the start of a weight-loss journey. Although the number of participants was small, analysis was strengthened by the large purchase volume. Potential misclassification during item matching cannot be ruled out but would likely affect both preinitiation and postinitiation purchases equally, minimizing systematic bias.

## Notes

Supplement.
Data Sharing Statement
