# Supplementary material for: Consumer Food Purchases After Glucagon-Like Peptide-1 Receptor Agonist Initiation
Source: JAMA Netw Open. 2026 Jan 26;9(1):e2555449. doi: 10.1001/jamanetworkopen.2025.55449 (PMC12836129; doi:10.1001/jamanetworkopen.2025.55449)
Supplement: Supplement. — Data Sharing Statement [file jamanetwopen-e2555449-s001.pdf]

Data Sharing Statement

Sørensen. Consumer Food Purchases After Glucagon-Like Peptide-1 Receptor Agonist Initiation. *JAMA Netw Open*. Published January 26, 2026.
doi:10.1001/jamanetworkopen.2025.55449

Data
Data available: No
